# Validity of the frame subtraction method in dynamic postural stability

**DOI:** 10.1186/s13102-022-00570-7

**Published:** 2022-09-26

**Authors:** Megumi Ota, Hiroshige Tateuchi, Takaya Hashiguchi, Karen Fujiwara, Ayano Sasaki, Kiseki Okumura, Noriaki Ichihashi

**Affiliations:** 1 Department of Preventive Physical Therapy, Human Health Sciences, Graduate School of Medicine, Kyoto University, 53 Kawahara-cho, Shogoin, Sakyo-ku, Kyoto, 606-8507 Japan; 2 Department of Physical Therapy, Human Health Sciences, Graduate School of Medicine, Kyoto University, 53 Kawahara-cho, Shogoin, Sakyo-ku, Kyoto, 606-8507 Japan; 3 Mixi Incorporated, Shibuya Scramble Square 36F, 2-24-12 Shibuya, Shibuya-ku, Tokyo, 150-6136 Japan; 4 Kansai Medical University Hospital, 2-3-1 Shin-machi, Hirakata, Osaka 573-1191 Japan

**Keywords:** Frame subtraction method, Motion analysis, Markerless system, Dynamic balance, Postural stability

## Abstract

**Background:**

The movement of targeted subjects can be calculated using the frame subtraction method. However, the validity of this evaluation method of dynamic postural stability has not been clarified yet. This study aimed to verify the validity of the evaluation method for jump landing using the frame subtraction score based on the ground reaction force (GRF).

**Methods:**

Twenty subjects performed single-leg jump landing, and their dynamic postural stability index (DPSI), medial‒lateral stability index (MLSI), anterior‒posterior stability index, and vertical stability index (VSI) were calculated from the GRF. Simultaneously, motion images were captured using digital video cameras in the sagittal and frontal planes. After the motion images were analyzed using the frame subtraction method, the frame subtraction scores in the frontal, sagittal, and combined planes were calculated. To confirm its validity, the relationship between the frame subtraction scores and GRF parameters was investigated using Pearson's correlation analysis.

**Results:**

The frame subtraction scores in the frontal and combined planes were significantly correlated with the DPSI, MLSI, and VSI (r = 0.46–0.75, *P* < 0.05).

**Conclusions:**

Therefore, the frame subtraction method could be applied to the evaluation of dynamic postural stability. Markerless systems are deemed useful in clinical practice.

## Background

In various sports, there is a high incidence of lower limb injuries, particularly at the ankle and knee joints [1, 2]. Patients with joint instability after ankle or knee ligament injuries tend to have diminished postural stability [3–7]. Single-leg jump landing is one of the motion tasks that evaluate dynamic postural stability for patients with joint instability after ankle or knee ligament injuries [8]. Patients with functional ankle instability had less postural stability during single-leg jump landing than in healthy subjects [9, 10]. Similarly, significant differences in dynamic postural stability during single-leg jump landing were observed between the injured and non-injured limbs of subjects who underwent unilateral anterior cruciate ligament (ACL) reconstruction [11]. Moreover, poor dynamic balance is a risk factor for knee and ankle ligament injuries [12, 13]. Therefore, to facilitate early return to sports and further prevent these leg injuries, dynamic postural stability should be assessed not only in the laboratory but also in the clinical and sports fields.

Currently, the dynamic postural stability index (DPSI) is used to evaluate dynamic balance through activities such as jump landing [7, 14]. The DPSI is indicated by the ground reaction force (GRF) that combines its components, such as the medial‒lateral stability index (MLSI), anterior‒posterior stability index (APSI), and vertical stability index (VSI). Since the DPSI is calculated by integrating the GRF components in three directions, it could comprehensively represent the ability to absorb the GRF in jump landing [15]. The DPSI is therefore a highly reliable and precise measure of dynamic postural stability [8]. In a previous study [11] targeted patients with ACL reconstruction, significant differences in dynamic postural stability were observed in the MLSI, APSI, VSI, and DPSI in jump landing between the surgical and non-surgical limbs. Additionally, an increased in APSI, VSI, and DPSI in jump landing was reported in patients with chronic ankle instability compared with those in healthy subjects [7]. However, to measure the DPSI, a force plate should be used. Measuring instruments, such as force plates, are expensive, and require a significant amount of time, technological skill, and knowledge to operate and analyze. Therefore, these measuring instruments are used to a limited extent in some special hospitals and laboratories. Since it is difficult to establish an environment in which postural stability can be evaluated with these measuring instruments in economic, technical, and physical aspects, objective and quantitative evaluation of dynamic balance has generally not been performed in most clinical and sports fields.

The frame subtraction method is widely used to detect moving objects in a sequence of frames from static cameras. In an environment where nothing moves, except for the targeted subject, the motions of the targeted subject, such as postural sway, can be quantified from the captured images using this method. Therefore, we hypothesized that this method could be applied to evaluate postural stability. Image analysis only requires standard digital video cameras available in the market, thereby reducing the cost. Moreover, this system is easier and more portable compared to conventional measuring instruments. Considering the above-mentioned advantages, it could be better applicable outside the laboratory. First, in our previous study [16], we verified the criterion-related validity of the frame subtraction scores and the center of pressure (COP) parameters during maintained single-leg standing. Results showed that the sum of the frame subtraction score in the frontal plane was significantly correlated with all COP displacements during single-leg standing, confirming the applicability of this method in evaluating static postural stability. However, studies applying the frame subtraction method to the evaluation of postural stability during dynamic balance tasks yet have not yet been conducted. Thus, this study aimed to verify the criterion-related validity of the evaluation of dynamic postural stability during single-leg jump landing using the frame subtraction method. We hypothesized the possibility of correlations between the frame subtraction scores: (1) in the frontal plane and MLSI, (2) in the sagittal plane and APSI, and (3) in the combined planes and DPSI.

## Methods

### Participants

The subjects were 20 healthy young people (10 males and 10 females; age, 21.8 ± 1.5 years; height, 165.3 ± 8.8 cm; weight, 59.1 ± 11.1 kg), and the G*Power V.3.1.9.4 program was used to determine the appropriate sample size before this study. With a single regression coefficient of > 0.6 (which is defined as strong) [17], a power of 80%, and an error level of 5%, 17 subjects were enrolled. Subjects with severe previous injuries to the extremities and trunk and balance-affecting nerve disorders were excluded. This study received approval from the ethics committee of Kyoto University Graduate School Faculty of Medicine (approval number: R1823), and all the subjects provided informed consent.

### Motion tasks

**Fig. 1:**
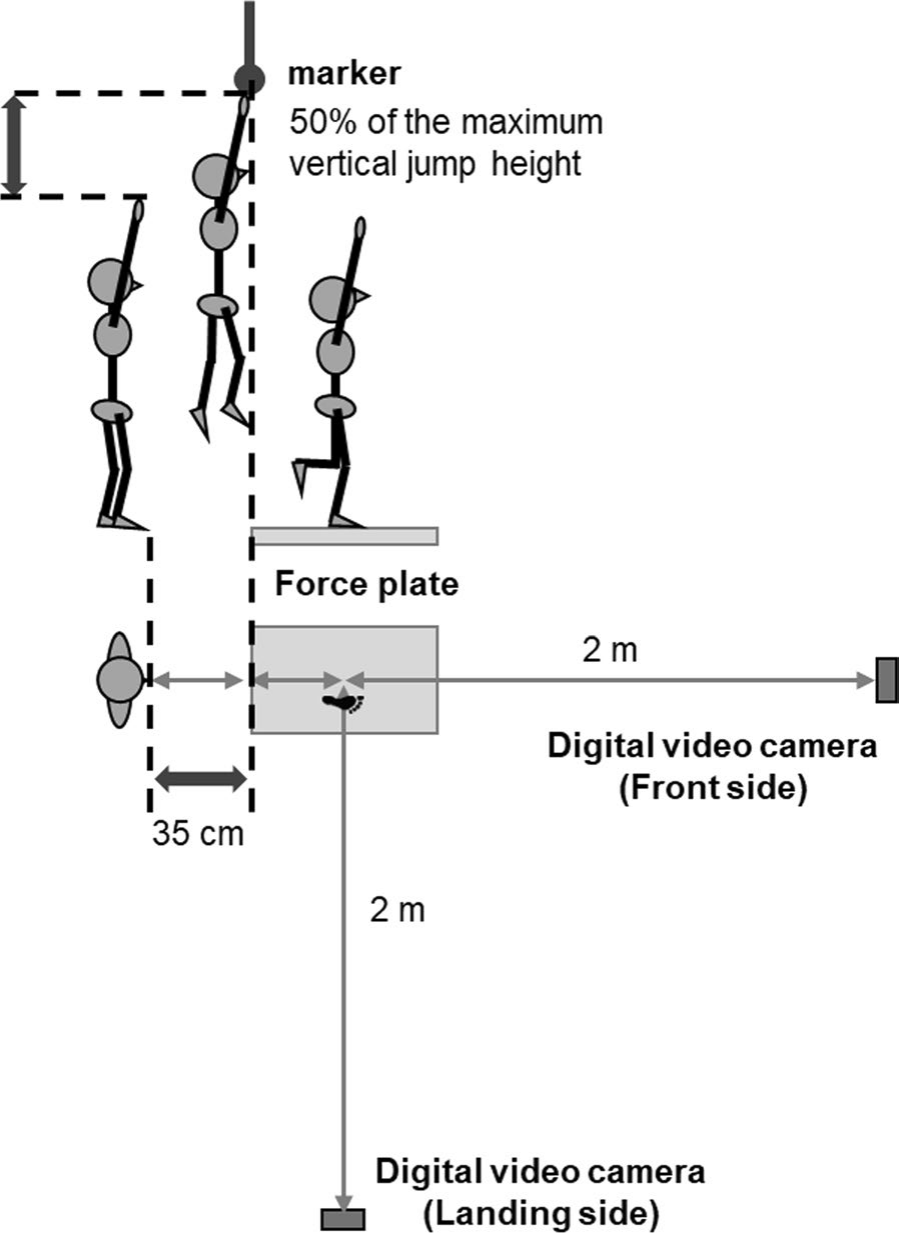
Experimental setup. The digital video cameras were set at 2.0 m in front and on the landing sides. The camera lens height was set at 87.5 cm. The starting position was 35 cm behind the edge of the force plate. The overhead marker was placed at a position equivalent to 50% of each participant's maximum vertical jump height

The motion task in this study was single-leg jump landing. The jump protocol was performed in accordance with the procedure mentioned in a previous study [18]. Figure 1 shows the experimental setup. First, after several familiarization jumps, the heights of the maximum vertical jump were measured; 50% of each subject's maximum vertical jump height was adjusted for them individually. Subsequently, the subjects stood 35 cm behind the edge of the force plate. They performed the motion task with their hand raised on the same side of their dominant leg and the other hand on their abdomen. Leg dominance was defined as the preferred leg for kicking a ball. They were instructed to jump anteriorly with both legs, touch an overhead marker placed at a position equivalent to 50% of each subject's maximum vertical jump height with their fingers on the same side of their dominant leg before landing on the force plate. Moreover, they were instructed to land on the center of the force plate with their dominant leg, stabilize as quickly as possible, and hold the position for 10 s after landing. After several practice trials, three successful trials were averaged for statistical analysis. Trials in which the participant could not jump higher than the instructed height or hold the instructed position for over 10 s after landing were excluded.

### Collection of data

#### The GRF parameters

The GRF parameters during single-leg jump landing were collected using a force plate (Kistler Japan Co., Ltd., Tokyo, Japan). The force plate data were sampled at a frequency of 1000 Hz [8] and passed through a zero-lag fourth-order low-pass Butterworth filter with a 20-Hz cutoff frequency [19]. Initial contact was defined as the instant the vertical GRF exceeded 10 N [11, 20], and the analysis interval was 3 s immediately after landing [8]. Then the DPSI, MLSI, APSI, and VSI were calculated using the formulas below [8]:

$$\begin{aligned} {\text{MLSI}} & = \sqrt {{{\sum {\left( {\frac{{0 - {\text{GRFx}}}}{{{\text{BW}}}}} \right)^{2} } } \mathord{\left/ {\vphantom {{\sum {\left( {\frac{{0 - {\text{GRFx}}}}{{{\text{BW}}}}} \right)^{2} } } {{\text{number}}\;{\text{of}}\;{\text{data}}\;{\text{points}}}}} \right. \kern-\nulldelimiterspace} {{\text{number}}\;{\text{of}}\;{\text{data}}\;{\text{points}}}}} \\ {\text{APSI}} & = \sqrt {{{\sum {\left( {\frac{{0 - {\text{GRFy}}}}{{{\text{BW}}}}} \right)^{2} } } \mathord{\left/ {\vphantom {{\sum {\left( {\frac{{0 - {\text{GRFy}}}}{{{\text{BW}}}}} \right)^{2} } } {{\text{number}}\;{\text{of}}\;{\text{data}}\;{\text{points}}}}} \right. \kern-\nulldelimiterspace} {{\text{number}}\;{\text{of}}\;{\text{data}}\;{\text{points}}}}} \\ {\text{VSI}} & = \sqrt {{{\sum {\left( {\frac{{0 - {\text{GRFz}}}}{{{\text{BW}}}}} \right)^{2} } } \mathord{\left/ {\vphantom {{\sum {\left( {\frac{{0 - {\text{GRFz}}}}{{{\text{BW}}}}} \right)^{2} } } {{\text{number}}\;{\text{of}}\;{\text{data}}\;{\text{points}}}}} \right. \kern-\nulldelimiterspace} {{\text{number}}\;{\text{of}}\;{\text{data}}\;{\text{points}}}}} \\ {\text{DPSI}} & = \sqrt {\frac{{\sum {\left( {\frac{{0 - {\text{GRF}}x}}{{{\text{BW}}}}} \right)^{2} } + \sum {\left( {\frac{{0 - {\text{GRFy}}}}{{{\text{BW}}}}} \right)^{2} } + \sum {\left( {\frac{{0 - {\text{GRFz}}}}{{{\text{BW}}}}} \right)^{2} } }}{{{\text{number}}\;{\text{of}}\;{\text{data}}\;{\text{points}}}}} \\ \end{aligned}$$

BW represents the body weight.

#### Frame subtraction method

In an environment where only the targeted subject moves, the differences between the images are caused by the motions of the subject. The algorithm for the frame subtraction method is as follows [21, 22]:

1. The absolute value of the subtraction between three consecutive images was to be calculated, and two subtraction images were to be created.
2. The logical product of two subtraction images was calculated, and a logical product image was created.
3. Binarization processing on the logical product image was performed.
4. The total value for each frame was calculated, which was determined as the frame subtraction score for that frame.

In other words, this subtraction based on the subject motions is used to obtain the frame subtraction score. We have established a program that processes images automatically. With our program, when we uploaded images to the cloud, the frame subtraction score was automatically calculated. The processing of motion images took about a few seconds to complete the calculation of the frame subtraction score. The motion images were recorded using digital video cameras both from the front and dominant leg (landing) sides of the subjects. The height from the floor surface to the lens was set at 87.5 cm, and the distance from the landing position to each camera was set at 2.0 m. The sampling rate was 60 Hz, and the pixel count was 130,000 pixels. First, the images were processed by the frame subtraction method; subsequently, the frame subtraction scores in the frontal and sagittal planes were quantified. Next, the sum of the frame subtraction score in the frontal and sagittal planes were determined the frame subtraction score in combined planes [16]. The initial contact was defined visually from the motion images. The analysis interval was 3 s immediately after landing. The maximum, sum, and root mean square (RMS) of the frame subtraction scores in each plane and combined planes were used as indicators of postural stability.

### Statistical analysis

Pearson's correlation coefficient was used to confirm the criterion-related validity between the frame subtraction scores and to measure the GRF parameters. The correlation coefficients (r) were determined as follows: r < 0.2, very weak; 0.2 ≤ r < 0.4, weak; 0.4 ≤ r < 0.6, moderate; 0.6 ≤ r < 0.8, strong; r ≥ 0.8, very strong [17]. Values of *P* < 0.05 were considered significant. The statistical analyses were performed using IBM SPSS Statistics, version 26 (IBM Japan Ltd., Tokyo, Japan).

## Results

**Fig. 2:**
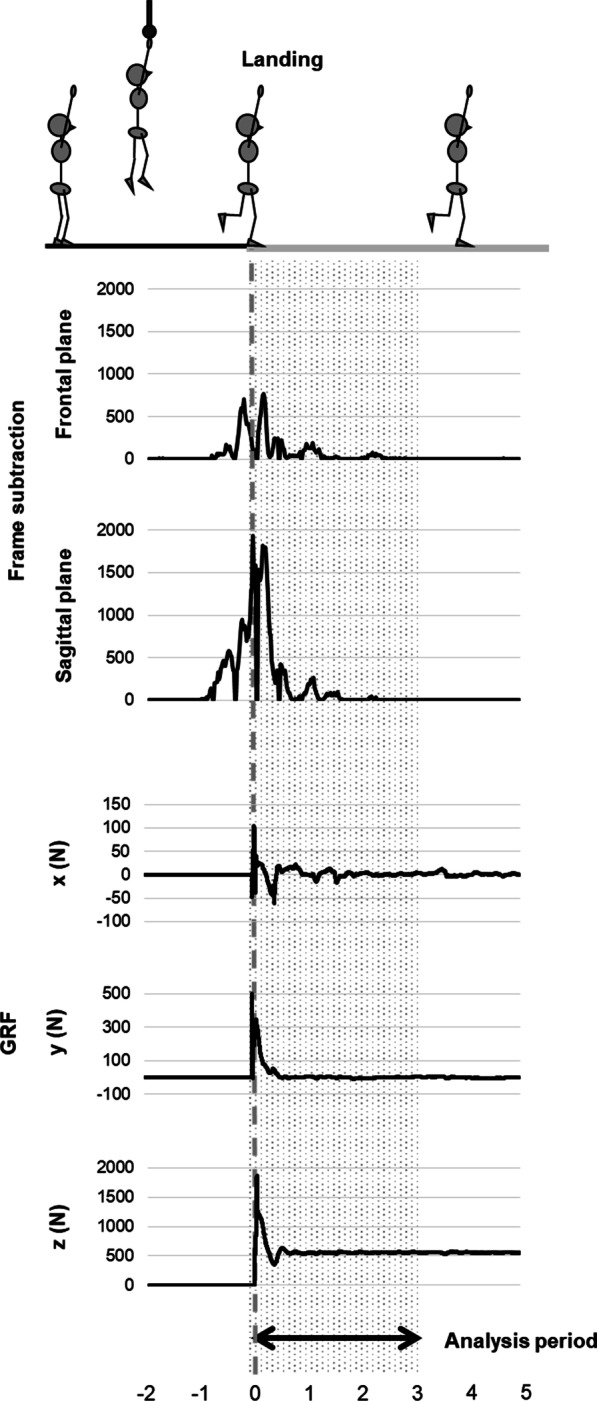
Typical examples of the frame subtraction scores and the GRF. GRF, ground reaction force; x, medial–lateral direction; y, anterior–posterior direction; z, vertical direction

**Fig. 3:**
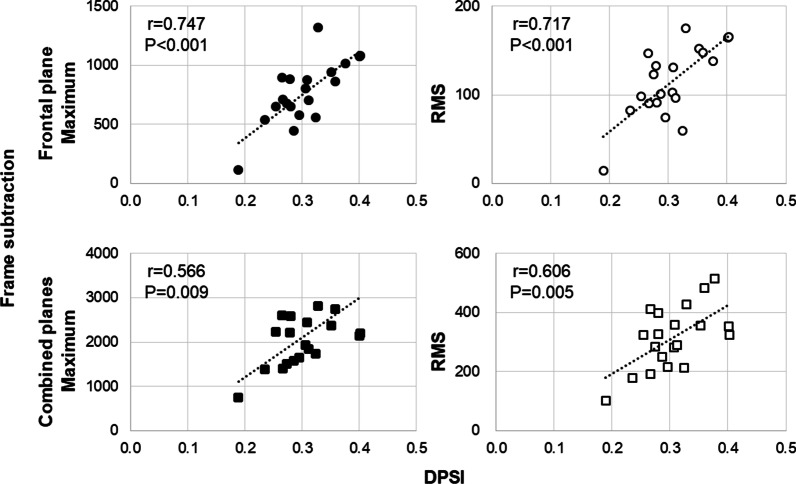
Scatter plots of the frame subtraction scores and the DPSI. DPSI, dynamic postural stability index; RMS, root mean square

Typical examples of the displacement of the GRF and the frame subtraction scores in the frontal and sagittal planes are shown in Fig. 2. The values for the GRF parameters and frame subtraction scores are described in Table 1. As shown in Table 2 and Fig. 3, significant moderate-to-strong correlations between the maximum and RMS of the frame subtraction scores in the frontal and combined planes and the DPSI. In particular, correlations were observed between the frame subtraction scores in the frontal plane and DPSI were over 0.7. Similarly, significant moderate correlations were observed between the frame subtraction scores in the frontal and combined planes and VSI. Additionally, significant correlations between the frame subtraction scores in the frontal plane and MLSI were observed. In other words, considering the frame subtraction scores in the frontal plane, significant moderate-to-strong correlations were found between the frame subtraction scores and GRF parameter except for APSI. Contrarily, significant correlations were absent between the frame subtraction scores in the sagittal plane and the GRF parameters, except for the RMS of the frame subtraction score and DPSI.

**Table 1:** The values for the GRF parameters and frame subtraction scores

Outcome	Mean (standard deviation)
*GRF parameters*	
DPSI	0.31 (0.05)
MLSI	0.03 (0.01)
APSI	0.10 (0.01)
VSI	0.30 (0.06)
*Frame subtraction scores*	
Frontal plane	
Maximum of the frame subtraction score	765.7 (259.7)
Sum of the frame subtraction score	7865.8 (3046.6)
RMS of the frame subtraction score	114.3 (39.5)
Sagittal plane	
Maximum of the frame subtraction score	1418.5 (718.1)
Sum of the frame subtraction score	12,935.2 (5770.1)
RMS of the frame subtraction score	210.0 (77.8)
Combined planes	
Maximum of the frame subtraction score	2148.2 (834.2)
Sum of the frame subtraction score	20,801.0 (8042.6)
RMS of the frame subtraction score	314.2 (102.2)

*GRF* ground reaction force, *DPSI* dynamic postural stability index, *MLSI* medial‒lateral stability index, *APSI* anterior‒posterior stability index, *VSI* vertical stability index, *RMS* root mean square

**Table 2:** Pearson's correlation coefficients between the frame subtraction score and the GRF parameter

	DPSI	MLSI	APSI	VSI
Frontal plane				
Maximum of the frame subtraction score	*r *= **0.747** *P* **< 0.001**	*r*** = 0.464** *P*** = 0.039**	*r* = − 0.253 *P* = 0.282	*r* **= 0.609** *P *** = 0.004**
Sum of the frame subtraction score	*r ***= 0.491** *P***= 0.028**	*r* **= 0.622** *P* **= 0.003**	*r* = − 0.069 *P* = 0.74	*r* **= 0.527** *P*** = 0.017**
RMS of the frame subtraction score	*r* **= 0.717** *P*** < 0.001**	*r*** = 0.592** *P* **= 0.006**	*r* = − 0.258 *P* = 0.272	*r***= 0.642** *P*** = 0.002**
Sagittal plane				
Maximum of the frame subtraction score	*r* = 0.432 *P* = 0.057	*r* = − 0.020 *P* = 0.932	*r* = − 0.365 *P* = 0.114	*r* = 0.387 *P* = 0.092
Sum of the frame subtraction score	*r* = 0.306 *P* = 0.190	*r* = 0.265 *P* = 0.259	*r* = − 0.085 *P* = 0.722	*r* = − 0.301 *P* = 0.196
RMS of the frame subtraction score	*r***= 0.466** *P* **= 0.039**	*r* = 0.142 *P* = 0.551	*r* = − 0.278 *P* = 0.235	*r* = 0.419 *P* = 0.066
Combined planes				
Maximum of the frame subtraction score	*r* **= 0.566** *P***= 0.009**	*r* = 0.106 *P* = 0.656	*r* = − 0.374 *P* = 0.104	*r***= 0.490** *P*** = 0.028**
Sum of the frame subtraction score	*r* = 0.405 *P* = 0.076	*r* = 0.426 *P* = 0.061	*r* = − 0.087 *P* = 0.716	*r* = 0.416 *P* = 0.068
RMS of the frame subtraction score	*r*** = 0.606** *P ***= 0.005**	*r* = 0.305 *P* = 0.191	*r* = − 0.303 *P* = 0.194	*r* **= 0.531** *P * **= 0.016**

*GRF* ground reaction force, *DPSI* dynamic postural stability index, *MLSI* medial‒lateral stability index, *APSI* anterior‒posterior stability index, *VSI* vertical stability index, *RMS* root mean square

Only statistically significant variables (*P* < 0.05) are shown in bold

## Discussion

This study is the first to apply the frame subtraction method to evaluate dynamic postural stability. We found significant positive correlations between the maximum and RMS of the frame subtraction score in the frontal and combined planes and the DPSI during single-leg jump landing. As hypothesized, correlations between the frame subtraction scores were observed in the frontal plane and MLSI, and between the frame subtraction scores on the combined planes and DPSI. However, contrary to our hypothesis, the frame subtraction scores in the sagittal plane did not correlate with APSI.

It is necessary to quantify the postural sway to evaluate the postural stability. The captured motion images using a digital video camera were processed by these technologies and used for objective evaluations of postural stability [23–25]. In previous studies using a markerless motion estimation technique, the center of mass (COM) trajectories during stable and unstable standing [24] and leg-up periods during single-leg standing [25] were measured. From the images captured with one digital video camera during balance tasks, the body parts with static postural sway can be estimated. Moreover, our previous study [16] has already demonstrated the criterion-related validity of the evaluation of postural stability during single-leg standing using the frame subtraction method. The sum of the frame subtraction score in the frontal plane was significantly correlated with the total length of COP displacements, RMS area, range in the anterior‒posterior direction, and range in the medial‒lateral direction [16]. However, these previous studies [16, 23–25] had focused on postural sway during the maintenance of static posture; whereas video image analysis, especially the frame subtraction method, has not yet been applied to dynamic balance evaluation. Although, some previous studies [26, 27] have evaluated dynamic balance using markerless motion estimation technique, these studies were on motion analysis during dynamic balance tasks. Moreover, a camera incorporating a depth sensor, such as Kinect, was used in these studies. Therefore, we verified the application for dynamic balance evaluation using images captured with one standard digital video camera available in the market.

Our results suggest that the frame subtraction score in the frontal and combined planes can indicate balance ability during single-leg jump landing. The DPSI is a composite of the MLSI, APSI, and VSI, and is sensitive to changes in all directions. Moreover, the precision and reliability of the DPSI are equivalent to or higher than those of the MLSI, APSI, or VSI only [8].Therefore, our study has focused on the DPSI within the measured GRF parameters. First, the DPSI did not correlate with the sum of the frame subtraction scores in any plane. The DPSI reflects the COM deceleration immediately after the jump landing, which indicates kinematic energy absorption [15]. In this study, the peaks of the frame subtraction scores were indicated immediately after single-leg jump landing. With an increase in the peak knee flexion angle, there would be low-impact forces [28], as the high-impact forces might be absorbed by remarkable joint motions. Therefore, the DPSI may be more strongly related to the maximum of the frame subtraction score. Additionally, since the DPSI is the RMS of the GRF normalized by body weight, it indicates convergence of continuous change in the GRF. Therefore, the DPSI may be related to the RMS of the frame subtraction scores, which indicates the convergence of continuous change in joint motions. The reason why all frame subtraction scores in the sagittal plane not showing any association to the GRF parameters may be because of individual differences in the posture control pattern in the sagittal plane during single-leg jump landing. Patterns with remarkable joint motions, such as hip and knee flexion‐extension, and patterns without remarkable joint motions, such as ankle strategy, can have these individual differences.

Although the images in the frontal and sagittal planes were analyzed in this study, only the frame subtraction scores in the frontal and combined planes were associated with the DPSI. Therefore, when evaluating single-leg jump landing, only images captured in the frontal plane may be sufficient for the evaluation of dynamic postural stability. Postural sway during single-leg jump landing may be possibly evaluated using only one digital video camera. The cost of evaluating postural stability using the frame subtraction method is low because it does not require any special measuring instruments. This indicates its beneficial application in several clinical and sports fields.

This study has several limitations. First, the features of the frame subtraction method may have caused limitations. Since all objects in motion were identified by this method, it is necessary to establish an imaging range with only the subject as the object in motion. Second, although jump landing is a common balance task in the clinical and sports fields [9–11], it has not yet been investigated whether the frame subtraction method is applicable to more dynamic balance tasks, such as cutting and turning, star excursion balance tasks, and single-limb squats commonly applied to patients with ACL and chronic ankle instability [29–32]. Therefore, further studies are required. We clarified that the frame subtraction method can be used to evaluate posture-maintaining balance tasks, such as single-leg standing [16] and jump landing. However, clarifications are required to measure its accuracy to detect balance disorders in patients compared to those in healthy subjects. In the future, it is necessary to verify our new proposed method in patients with static or dynamic balance disorders.

## Conclusions

This study verified the criterion-related validity of the frame subtraction method based on the evaluation of dynamic postural stability. Regarding single-leg jump landing, the frame subtraction scores in the frontal plane were significantly correlated with the DPSI, MLSI, and VSI. Although two digital video cameras were used in this study, it is possible to measure postural sway and evaluate the dynamic postural stability using just a single frontal video camera. Since the frame subtraction method can reduce costs and time because of its markerless system, it can be an alternative to conventional measuring instruments, such as force plates.

## Data Availability

The datasets generated and/or analyzed during the current study are not publicly available due to ethical restrictions, but are available from the corresponding author on reasonable request.

## Abbreviations

ACL: Anterior cruciate ligament
DPSI: Dynamic postural stability index
GRF: Ground reaction force
MLSI: Medial‒lateral stability index
APSI: Anterior‒posterior stability index
VSI: Vertical stability index
COP: Center of pressure
BW: Body weight
RMS: Root mean squares
COM: Center of mass
